# Direct Methanol (or Ethanol) Fuel Cell as Enzymatic or Non-Enzymatic Device, Used to Check Ethanol in Several Pharmaceutical and Forensic Samples

**DOI:** 10.3390/s18113596

**Published:** 2018-10-23

**Authors:** Mauro Tomassetti, Riccardo Angeloni, Sergio Marchiandi, Mauro Castrucci, Maria Pia Sammartino, Luigi Campanella

**Affiliations:** Department of Chemistry, “La Sapienza” University of Rome, 00185 Rome, Italy; r.angeloni_08@libero.it (R.A.); sergio_marchiandi@virgilio.it (S.M.); mauro.castrucci@libero.it (M.C.); mariapia.sammartino@uniroma1.it (M.P.S.)

**Keywords:** DMFC, enzymatic and non-enzymatic DMFC, ethanol analysis, drugs, serum, saliva, imipenem test

## Abstract

It was already demonstrated by our research group that a direct catalytic methanol (or ethanol) fuel cell (DMFC) device can be used also for analytical purposes, such as the determination of ethanol content in beverages. In the present research we extended the application to the analysis of several ethanol-based pharmaceutical products, i.e., pharmaceutical tinctures (dyes) and disinfectants. In recent work we have also shown that the use of alcohol dehydrogenase enzyme as a component of the anodic section of a direct catalytic methanol (or ethanol) fuel cell significantly improves the performance of a simple DMFC device, making it more suitable to measure ethanol (or methanol) in real samples by this cell. At the same time, we have also shown that DMFC can respond to certain organic compounds that are more complex than methanol and ethanol and having R(R’)CH-OH group in the molecule. Firstly, pharmaceutical dyes were analyzed for their ethanol content using the simple catalytic DMFC device, with good accuracy and precision. The results are illustrated in the present paper. Additionally, a detailed investigation carried out on commercial denatured alcoholic samples evidenced several interferences due to the contained additives. Secondly, we hypothesized that by using the enzymatic fuel cell it would be possible to improve the determination, for instance, of certain antibiotics, such as imipenem, or else carry out determinations of ethanol content in saliva and serum (simulating forensic tests, correlated to drivers “breath test”); even if this has already been hypothesized in previous papers, the present study is the first to perform them experimentally, obtaining satisfactory results. In practice, all of the goals which we proposed were reached, confirming the remarkable opportunities of the enzymatic (or non-enzymatic) DMFC device.

## 1. Introduction

Researches of different types have been performed in the past by some authors with the aim of using different fuel-cell-based devices for analytical [1,2,3,4,5,6,7] (and energetic [8,9,10,11,12,13,14,15,16,17,18,19,20,21,22,23,24]) purposes. These researches have effectively shown the possibility of this kind of application, but only by means of particular and complex types of fuel cell, not useful for common application to real samples. However, our research group recently performed the first researches of this type, by using a simple and suitable direct methanol fuel cell (DMFC) [25,26]. In a further study [27], the performances of our fuel cell were made more efficient for the determination of ethanol by immobilizing an enzyme into the device [27]. Our group has continued this analytical research, devoted to enzymatic (or non-enzymatic) DMFC applications for analytical purposes. New unpublished results, reported in the present paper, concern several further analytical applications in pharmaceutical and forensic fields. Firstly, we continued to investigate the possibility of checking, in a simple and inexpensive way, the alcohol content of some drugs available in drugstores. By using a non-enzymatic DMFC device, the ethanol content of several pharmaceutical dyes was easily checked. The obtained results were compared both with the ethanol content declared by the producer and with the obtained data, by analyzing the same samples using a conventional amperometric catalase enzyme sensor, recently prepared in our laboratory and already used to validate results obtained using DMFC device for ethanol determination in beverages [26]. It was also attempted to utilize the fuel cell to validate the ethanol content of denatured commercial ethanol, usually available in drugstores. In this case, however, great interferences occurred due of the presence of red dye (Reactive Red 120) and denatonium benzoate, both of which are contained in this kind of commercial product. The best opportunity was the determination of a particular antibiotic (imipenem), which is an active agent having a -C(CH_3_)H-OH functional group in its molecule, contained in some pharmaceutical formulations. In this case, we used the enzymatic DMFC device for the measurement. The enzyme introduced in the anodic cell section gave the possibility of improving the analytical performances of the catalytic fuel cell, as, for instance, in the case of imipenem, and as was observed in the case of ethanol [27]. To this purpose, the alcohol dehydrogenase enzyme was immobilize in a dialysis membrane small bag located in the anodic area of the fuel cell. The enzyme with its catalytic action increased the sensitivity of the method and dramatically reduced the response time of the cell. Owing to the presence of the same enzyme in the fuel cell, it was also possible to evaluate the ethanol content in some human saliva and serum samples, a measurement that may be of interest to determine the alcohol level in specific human biological fluids usually checked in support of rapid “breath testing” of drivers.

## 2. Materials and Methods

### 2.1. Apparatus

**Figure 1:**
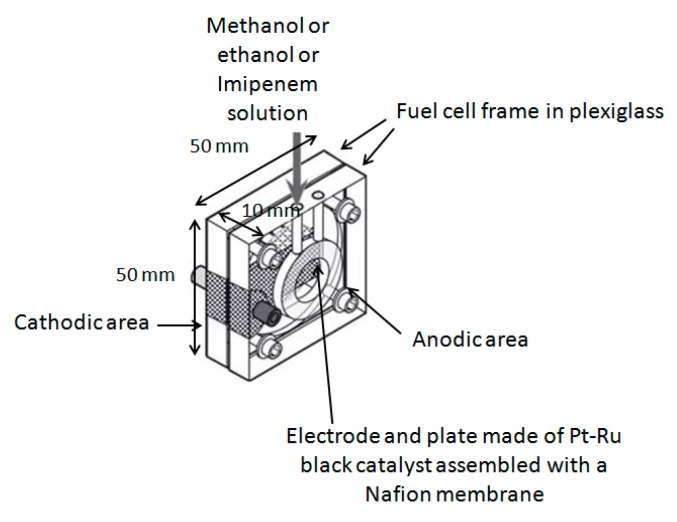
Direct catalytic fuel cell used for analytical purpose, purchased from “Fuel Cell Store”, H-Tec model F111.

The small catalytic direct methanol or ethanol fuel cell was obtained from Fuel Cell Store (College Station, TX, USA), originally constructed with the aim of obtaining energy from methanol or ethanol, and optimized by us for analytical purposes. The dimensions of the fuel cell (Figure 1) (weight: 100 g) were 50 mm × 50 mm × 10 mm. The electrode area was about 4 cm^2^ and the maximum generated power was about 10 mW. The fuel cell frame was made of Plexiglas^®^, while the electrode end plate was of a Pt-Ru black catalyst assembled with a Nafion™ membrane. The alcohol dehydrogenase enzyme (from *Saccharomyces cerevisiae* E.C.1.1.1.1, CAS: 9031-72-5) was supplied by Sigma-Aldrich (Milan, Italy).

### 2.2. Analyzed Samples

Analyzed dyes were officinal preparations purchased in drugstore, whose ethanol contents declared by the producers were in the interval 54.92–89.78% by volume. These pharmaceutical preparations have different healing properties depending on the contained traces of different vegetable extracts. Additionally, analyzed denatured ethanol samples were purchased in common drugstore, while imipenem antibiotic was obtained from hospital pharmacy. Standard human serum samples were purchased from Sigma (St. Louis, MO, USA), while saliva samples were of the authors of this paper. As such, all samples were directly analyzed only after simple dilution, if necessary, by distilled and deionized water. Diluted values are respectively indicated in the tables reported in the “Results and Discussion” section.

### 2.3. Electrochemical Background

The anodic, cathodic, and global reactions of the oxidation of methanol or ethanol to carbon dioxide which occur in the DMFC device are the following:
(For methanol)Anodic reaction: CH_3_OH + H_2_O ⟶6H^+^ + 6 e^−^ + CO_2_(oxidation)Cathodic reaction: 3/2 O_2_ + 6H^+^ + 6 e^−^ ⟶3H_2_O(reduction)Global reaction: CH_3_OH + 3/2 O_2_ ⟶2H_2_O + CO_2_(redox)(For ethanol)Anodic reaction: C_2_H_5_OH + 3H_2_O ⟶12H^+^ + 12 e^−^ + 2CO_2_(oxidation)Cathodic reaction: 3 O_2_ + 12H^+^ + 12 e^−^ ⟶6H_2_O(reduction)Global reaction: C_2_H_5_OH + 3 O_2_ ⟶3H_2_O + 2CO_2_(redox)

### 2.4. Fuel Cell Measurement, Apparatus, and Calibration Curves

**Figure 2:**
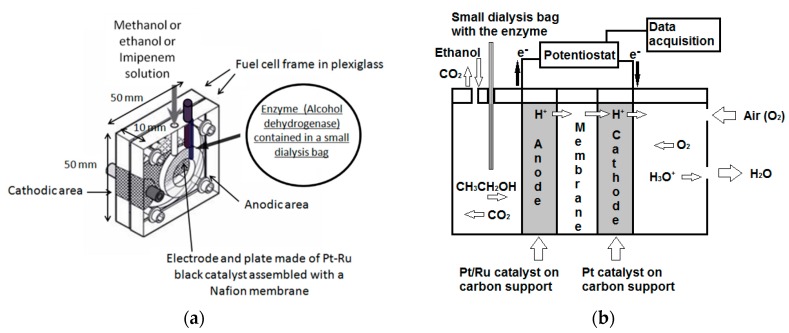
(**a**) Enzymatic fuel cell; (**b**) Detailed scheme of the enzymatic fuel cell (side view).

In a previous study [25], the first experiments were performed by fuel cell operating at open circuit voltage (OCV), testing the e.m.f. (electro motive force), by a digital multimeter, with high input impedance (about 10 GΩ), which allowed measurements. Additionally, in another study [27], the measurement was proved to be more suitable for working in potentiostatic format mode [27]. In the latter case, the supplied current (SC) through the cell was measured. To this end a PalmSens potentiostat (mod. EmStat, PalmSens BV, Randhoeve 221, 3995 GA Houten, The Netherlands) was used, connected to a PC running PSTrace ver. 4.6. software for data recording and processing. The fuel cell anode, as working electrode, was connected to the EmStat, while the fuel cell cathode and the counter electrode were connected to the EmStat reference. Before current measurement, the EmStat automatically measured the OCV value for about 200 s, and the anode potential was then set to a value 100 mV lower than the OCV value, that is, at the “Optimized Anodic Potential” value (OAP) previously experimentally established [25]. Although complex enzymatic fuel cell devices are reported in the literature [28], in our enzymatic DMFC device alcohol dehydrogenase was simply added to the anodic section of the fuel cell by means of a small dialysis bag, dipped into sample solution contained in the cell, as shown in Figure 2a. For the measurements in the presence of alcohol dehydrogenase (AD), a weighed quantity of this enzyme, i.e., 5 mg of alcohol dehydrogenase, was placed in a very small dialysis membrane cylindrical bag (3.5 mm in diameter and 3.5 cm in height) together with a drop of phosphate buffer. After carefully positioning a rigid plastic stick into the dialysis membrane (Sigma D-9777, Milan, Italy) bag, a sort of cylindrical stiff bag was obtained, which was sealed at the top, inside of which was contained the mush of the enzyme. The bag was placed into the anode area of the fuel cell (see Figure 2b) before the measurement. The successive measurement format was the same as that used for the non-enzymatic fuel cell: before each measurement, the fuel cell was washed with 0.5% V/V water–ethanol solution, and then carefully washed several times with distilled water. Subsequently, the fuel cell was filled with the solution to be analyzed (i.e., 2 mL) and closed to prevent evaporation of the alcohol. Measurements started after conditioning the system for about 60 s. The SC was then continually monitored until a steady state was reached. At this point, the supplied current was read off and correlated with the ethanol concentration in the fuel cell solution. All the measurements performed in the present research were carried out by using the potentiostatic mode, which was also adopted to record calibration straight lines both for the enzymatic and non-enzymatic catalytic fuel cell, by using several ethanol–water standard mixtures with increasing ethanol concentration.

### 2.5. Conventional Biosensor Method

**Figure 3:**
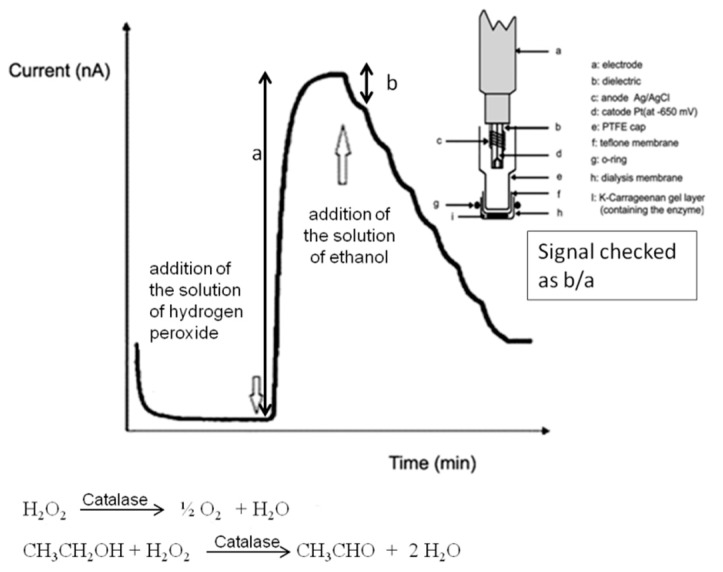
Functioning of conventional enzymatic amperometric biosensor used for data comparison.

In the literature there are several conventional methods for ethanol determination, i.e., titrimetrics [29] and instrumentals, for instance chromatographics [30,31,32,33,34] or spectrophotometrics [35,36,37,38,39], and in recent years also sensors-biosensor methods [40,41,42,43,44]. Our research group has recently also developed different methods for ethanol determination by using conventional biosensors. The more suitable of these conventional methods was based on an amperometric enzymatic biosensor using catalase, which was well standardized and was validated several times in previous papers [45,46,47] comparing the results also with HPLC methods [45]; consequently, this typical traditional method of proven reliability was chosen and used to compare and validate results of the present DMFC method in the application to the same samples. The detailed description of this amperometric enzymatic biosensor and the operative assembly (which is shortly illustrated in Figure 3), have been reported in previous papers [46,47].

## 3. Results and Discussion

In Table 1, the main analytical data obtained by both the simple fuel cell and the enzymatic fuel cell for ethanol determination (in the potentiostatic mode) are summarized and compared.

It can be observed that the linearity range and LOD (Limit of Detection) values are better for the enzymatic fuel cell and, above all, that the response time is short enough for the latter device, while the life time of the device is longer for the more inexpensive and simple non-enzymatic catalytic fuel cell. By using the simple DMFC device, the ethanol content of several pharmaceutical dyes were determined for a fast, simple, and cheap control of these kind of products. The main results are given in Table 2.

The obtained data are compared, in the same Table 2, both with the ethanol content data declared by the producer and with data obtained by analyzing the same samples by using the conventional amperometric catalase enzyme sensor as described in the previous paragraph. Table 2 shows the analytical validity of the values obtained with the fuel cell; if they are compared to the nominal values, the agreement is always of the same order as in the case of the test performed by the conventional catalase biosensor. On the other hand, the results of the F-test, shown in Table 3, show the precision for the two methods of analysis is always “not significant”, except in one case.

While in the present research we analyzed several pharmaceutical dyes, almost accidentally, we adopted the fuel cell to determine the more common based ethanol pharmaceutical product, i.e., the “disinfectant (or denatured) ethanol”. Curiously, we found that the values of this product were markedly lower than the expected ones, as shown in Table 4.

Table 4, presents a comparison between the values (expressed as percentage by volume) in three different commercial denatured ethanol samples purchased in drugstores, experimentally found by the simple DMFC device, and the nominal (% by volume) concentrations values claimed on the label. The values determined by DMFC were consistently lower than the nominal ones. To check if the ethanol content was the same as the claimed value, and if therefore this difference was to be attributed to the analytical method used, the considered denatured alcohol samples were simultaneously analyzed by catalase enzymatic biosensor method, i.e., a conventional method several times well validated in previous papers [27,45,46,47] (comparing it also with chromatographic tests [45]). The obtained results are displayed in Table 4. The data prove that the even lower ethanol content, which in all cases was highlighted by the fuel cell, must surely be attributed to the fuel cell experimental method. We therefore considered the hypothesis that additives present in the denatured alcohol could exert a noticeable interference, lowering the sensitivity of the fuel cell to ethanol. Information about disinfectant ethanol composition is not abundant; usually, the composition of denatured alcohol is not reported in detail on the label (particularly concerning the effective percentage of all the components of the mixture), because sometimes this is covered by patent. The values reported in the literature are generally the following: for denatured EtOH samples, a content not less than 83% by volume has been prescribed for “disinfectant ethanol” by the G.U. European (374/42 of 22 December 2004 i.e., the modified of Commission Regulation No 3199/93) [48]. Moreover, various other substances are added to commercially so-called “denatured alcohol”. These additions are usually: firstly, b-denatonium benzoate, in addition to a red dye (C.I. Red 24, C.I. 18208), or similar (C.I. Red 120), and lastly thiophene and methyl ethyl ketone. Therefore, all these substances were individually tested by us. We experimentally checked whether, individually, these compounds were able to give any response by themselves by using fuel cell, or if they were able to alter the response of the fuel cell to ethanol. The experimental results indicated that the b-denatonium benzoate, the red dye (C.I. Red 120), thiophene, and methyl ethyl ketone give practically no signal (at least at the concentration of about 10^−3^ mol·L^−1^), but all bring to lower responses of the fuel cell to ethanol, if the latter was also present in the cell. In conclusion, a comparison with reference analytical data, found both by using the conventional amperometric biosensor and official nominal values, confirmed that additives contained in denatured ethanol give interferences, lowering the response of the fuel cell to ethanol; this is probably because these components, especially denatonium, or Red Dye 120, can complicate the catalyst (ruthenium ions), as reported in the literature for compound of the same type [49]. Other successive applications have been performed on different kind of complex-relevant samples, by measuring the SC again, although in this case using the enzymatic device. In fact, in a previous study [27], when simple ethanol solutions were measured it was possible to demonstrate that, if alcohol dehydrogenase was introduced in the fuel cell, this enzyme was able to produce a small increase in the sensitivity of the method. In more detail, it can fasten the ethanol breakdown process [27] and therefore enhance the analytical performances of the fuel cell, as can be seen from the response time reported in Table 1.

**Figure 4:**
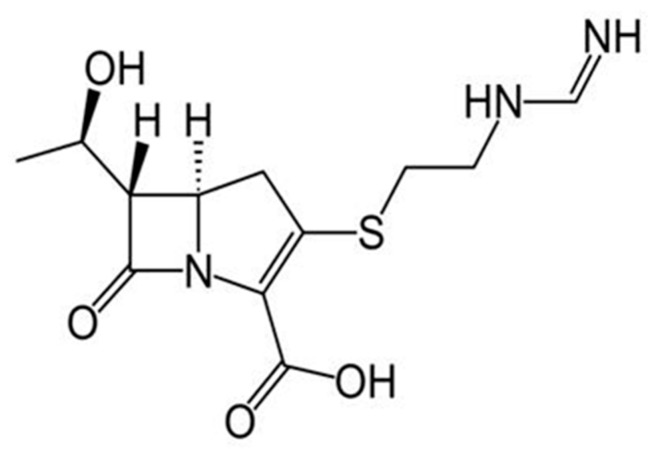
Structural formula of imipenem.

**Figure 5:**
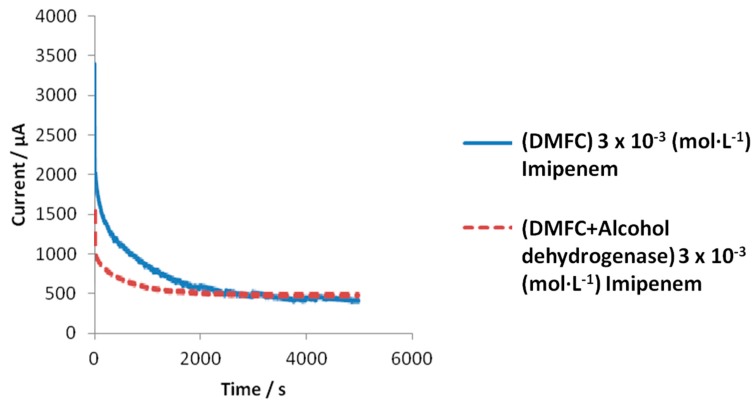
Comparison of supplied current (SC) vs. time of the enzymatic (dashed line) and non-enzymatic (bold line) fuel cell both containing a 3 × 10^−3^ mol·L^−1^ solution of imipenem.

Table 1 shows that the enzyme alcohol dehydrogenase dramatically reduces the response time of the cell to ethanol, which is of extreme importance for the analytical applications. We therefore considered it useful to check if, with the new enzymatic fuel cell, the same benefits already obtained in the case of simple ethanol sample analysis could also be obtained in the case of the analysis of a particular antibiotic, i.e., imipenem (see Figure 4). To this purpose, in Figure 5 a comparison is displayed between the experimental response behavior to imipenem, both of the enzymatic (alcohol dehydrogenase) and non-enzymatic DMFC devices, in potentiostatic mode at Optimized Anodic Potential applied (OAP); in both cases, the SC values vs. time were plotted until the steady state, for a typical imipenem concentration.

**Figure 6:**
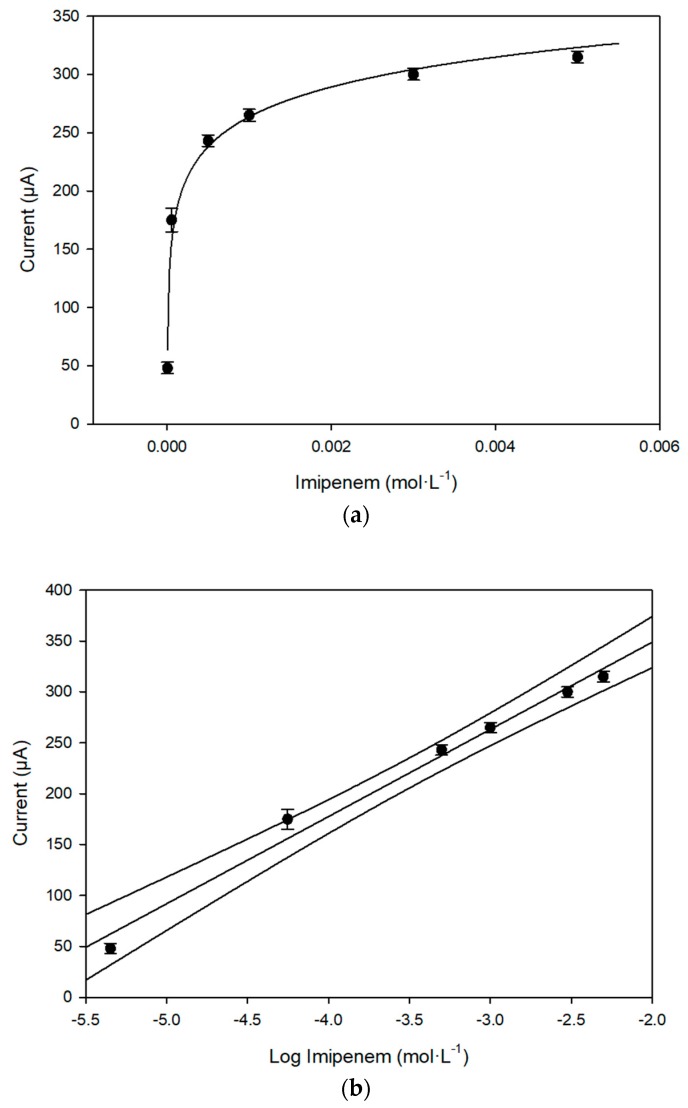
(**a**) Response to increasing imipenem concentration of the enzymatic (alcohol dehydrogenase) fuel cell (potentiostatic mode, at OAP); (**b**) Corresponding calibration curve, in semilogarithmic scale, to imipenem concentration of the enzymatic (alcohol dehydrogenase) fuel cell (potentiostatic mode, at OAP).

It can be observed immediately that the current reaches the steady state more rapidly in the presence of the enzyme alcohol dehydrogenase than when this enzyme is absent. In Figure 6a,b, the behavior of the response of the enzymatic fuel cell, on increasing imipenem concentration, and the corresponding calibration curve, in a semilogarithmic scale, are reported.

In Table 5, a comparison of the main analytical data, including the equation of the calibration curves to imipenem, by using enzymatic or non-enzymatic fuel cell, is reported.

It can be confirmed that on analyzing imipenem, the introduction of the enzyme significantly reduces the response time of the device. Furthermore, in Table 6, some selectivity data related to other types of antibiotics are reported. The selectivity of the fuel cell results is undoubtedly good.

Additionally, using the enzymatic fuel cell, an application was carried out on a relevant pharmaceutical formulation containing imipenem, by determining the content of this antibiotic in pharmaceutical products. Data obtained by applying the standard addition test method are reported in Table 7.

It can be concluded that the recovery data are acceptable. It was also ascertained that the fuel cell method is robust, as small variations of parameters such as temperature, pH, ionic strength, etc., do not appreciably influence the results of the measurements. Finally, a forensic application of the enzymatic fuel cell was tried. At present, there are some ethyl tests for the saliva of drivers available, and it was also established [50,51] that the alcohol concentration in saliva is comparable to that in blood or serum. For this purpose, the colorimetric test [52,53] is the most applied. It is usually considered as qualitative, at most semi-quantitative, as it is affected by many interferences, such as those from ascorbic acid, polyphenols, uric acid, oxalic acid, bilirubin, tannic acid, mercaptans, and generally strong oxidants [53]. This colorimetric test it is based on the following reactions:

(1) $${\mathrm{CH}}_{3}{\mathrm{CH}}_{2}\mathrm{OH}+O_{2}\overset{\mathrm{AlcoholOxidase}}{\to }{\mathrm{CH}}_{3}\mathrm{CHO}+H_{2}O_{2}$$

(2) $$H_{2}O_{2}+\mathrm{Tetramethylbenzidine}\overset{\mathrm{Peroxidase}}{\to }\mathrm{Green}\;\mathrm{Dye}+H_{2}O$$

The ethanol detection range usually falls between 0.02% (light green) and 0.30% (gray-green) (i.e., between about 4.3 × 10^−3^ mol·L^−1^ and 5.4 × 10^−2^ mol·L^−1^); therefore, as the linearity range of our enzymatic fuel cell, between about 5 × 10^−4^ mol·L^−1^ and 6 × 10^−1^ mol·L^−1^, widely covers the whole range of the current colorimetric test, it was decided to simulate ethyl test, with the enzymatic fuel cell, by determining the ethanol content both in saliva and in human serum samples, to which ethanol was added until final concentrations of 1.8 × 10^−3^ mol·L^−1^ and 1.0 × 10^−2^ mol·L^−1^, respectively, were reached; this means practically that these enzymatic fuel cell tests were carried out with biological samples having about the lowest and the highest respective concentrations provided usually also by ethyl-colorimetric test. The first results obtained are summarized in Table 8.

Data in Table 8 prove an acceptable repeatability of the measurements, while the *t*-test shows that the difference between nominal and experimental values is always insignificant. Therefore, although still requiring further investigations, the method is accurate enough.

## 4. Conclusions

The present research can be considered as belonging to the research field reported in the literature concerning the development of fuel cells [1,2,3,4,5,6,7,8,9,10,11,12,13,14,15,16,17,18,19,20,21,22,23,24] and biofuel cells [28,54,55,56,57] for several purposes. This study positively extended applications to several pharmaceutical products (dyes) by a simple DMFC catalytic device (already performed on several alcoholic beverage samples [25,26]). The application to test the ethanol content of denaturated alcohol failed. These analytical results have been validated, comparing them with those obtained on the same samples, using a typical conventional method [45,46,47], but previously very well validated, chosen therefore as well representative of the traditional methods reported in the literature. Moreover, in this research, it was firstly demonstrated that the fuel cell can be useful to determine the ethanol content in several relevant pharmaceutical formulations, as dyes, with sufficient precision, accuracy, and robustness; secondly it was demonstrated that the enzymatic device can be also utilized to test other particular more complex organic molecules of pharmaceutical interest, such as the antibiotic imipenem; lastly, it was demonstrated that the enzymatic fuel cell seems to be useful for certain analytical forensic purposes, i.e., the measurement of ethanol in saliva and serum. In conclusion, owing to the low cost, the very low encumbrance of the cell and of the measurement system [25], and the high sensitivity and the short response time, achieved by adding enzyme alcohol dehydrogenase to the anodic section, this small enzymatic DMFC device [27] can be proposed as a suitable tool for several simple and fast analytical tests to be easy applied also in situ, with a few additional precautions [25,26,27].

## Figures and Tables

**Table 1:** Comparison of the main analytical features of the simple catalytic fuel cell and catalytic-enzymatic fuel cell used in this study, on determining ethanol.

Method	Linearity Range (mol·L^−1^)	LOD (mol·L^−1^)	Life Time	Response Time
Fuel cell potentiostatic mode at OAP	1.0 × 10^−3^–4.0 × 10^−2^	8.0 × 10^−4^	Several weeks	≈55 min
Enzymatic (alcohol dehydrogenase) fuel cell potentiostatic mode at OAP	5.0 × 10^−4^–6 × 10^−1^	2.0 × 10^−4^	≥2 weeks	≤20 min

**Table 2:** Results of the analysis of different pharmaceutical dyes using the simple direct fuel cell and conventional catalase biosensor. Comparison among the nominal values after dilution to 1:1000 and the values found with both fuel cell and catalase biosensor.

Different Dyes Sample Number and Ethanol Nominal Content as % V/V Value	Ethanol Nominal Value (as mol·L^−1^) after Dilution 1:1000 (a)	Ethanol content (mol·L^−1^)			
		Fuel Cell RSD% ≤ 10 (n = 3) (b)	(b−a/a)%	Catalase Biosensor RSD% ≤ 15 (n = 3) (c) (c−a/a)%	(c−a/a)%
(1) 88.62	0.0152	0.0139	−8.6	0.0154	+1.3
(2) 64.71	0.0111	0.0106	−4.5	0.0114	+2.7
(3) 54.92	0.00942	0.0095	+0.8	0.0096	+1.9
(4) 89.78	0.0154	0.0163	+5.8	0.0149	−3.2
(5) 64.71	0.0111	0.0113	+1.8	0.0114	+2.7

**Table 3:** Results of F-test (“Fuel cell”—catalase biosensor): two sides, ν ”fuel cell” = ν biosensor = 4−1 = 3, p = 95%.

Sample	F-exp	F-critic	Result of the Test
(1)	2.367	15.44	Not significant
(2)	2.890		Not significant
(3)	225.0		Significant
(4)	4.592		Not significant
(5)	2.388		Not significant

**Table 4:** Comparison of data found for denatured ethanol by using both fuel cell and catalase biosensor.

Denatured Alcohol Sample n.	Nominal Values % V/V	EtOH Value Found by Catalase Biosensor (V/V) (RSD% ≤ 5)	EtOH Value Found by Fuel Cell % (V/V) (RSD% ≤ 5)
1	~70	71.2	55.3
2	~70	70.0	57.0
3	~95–96	90.5	61.2

**Table 5:** Main data of the analysis of imipenem by fuel cell in the absence and the presence of alcohol dehydrogenase enzyme in the anodic zone of the fuel cell.

	Non-Enzymatic	Enzymatic
Regression equation (Y = µA., X = mol·L^−1^)	Y = 37.6 (±3.3) logX + 481 (±27)	Y = 38.6 (±3.1) logX + 536 (±26)
Linearity range (mol·L^−1^)	(6.0 × 10^−6^–6.0 × 10^−3^)	(5.0 × 10^−6^–5.0 × 10^−3^)
R^2^	0.9820	0.9753
Pooled SD	6.0	6.2
LOD	5.0 × 10^−6^	5.0 × 10^−6^
RSD%	2.0	2.8
Response time (min)	≈90	≈20–25

**Table 6:** Selectivity data for fuel cell vs. several different antibodies.

Antibiotics	Response by Fuel Cell to Several Antibiotic RSD% ≤ 7.0. Response to Imipenem Checked as 100%
Imipenem	100.0
Penicillin G	0.00
Ampicillin	0.00
Amoxicillin	0.00
Cefalotin	0.00
Fosfomicin	0.00
Rifamicin	0.00

**Table 7:** Recovery test for imipenem in pharmaceutical formulation by enzymatic fuel cell.

Pharmaceutical Matrix	Imipenem Concentration in the Sample of Pharmaceutical Formulations Diluted (1:100) Before Spiking (mol·L^−1^)	Imipenem Concentration Added to the Spiked Diluted Samples (mol·L^−1^)	Total Concentration of the Antibiotic Contained in the Spiked Diluted Samples (Nominal Value) (mol·L^−1^)	Total Antibiotic Concentration in the Spiked Diluted Samples (Experimental Value) (mol·L^−1^) (n = 3) (RSD ≤ 5)	% Recovery (RSD% ≤ 5) (n = 3)
Pharmaceutical formulation containing imipenem	2.85 × 10^−3^	1.00 × 10^−3^	3.85 × 10^−3^	3.70 × 10^−3^	96.1

**Table 8:** Determination of ethanol content in human saliva and serum samples spiked with ethanol by using enzymatic (alcohol dehydrogenase) catalytic DMFC device, and results of *t*-test (each value is the mean of three determinations).

Sample n.	Type	Ethanol Content (Nominal Value) (mol·L^−1^) (a)	Ethanol Content Using Enzymatic Fuel Cell (Experimental Value) (mol·L^−1^) (b)	SD (mol·L^−1^) (n = 3)	Δ% = [(b−a)/b]%	Two Sides *t*-Test: p = 95%, ν = 3−1 = 2		
						t_exper._	t_critic_	Results of *t*-Test
1	Saliva	0.0103	0.0114	±0.0015	+10.6	1.270	4.303	N.S.
2	Saliva	0.0018	0.0016	±0.0002	−11.1	−1.732	4.303	N.S.
3	Serum	0.0103	0.0114	±0.0015	+10.6	1.270	4.303	N.S.
4	Serum	0.0018	0.0017	±0.0002	−5.6	−0.866	4.303	N.S.

N.S. = not significant.
